# Predicting the risk and timing of major mood disorder in offspring of bipolar parents: exploring the utility of a neural network approach

**DOI:** 10.1186/s40345-021-00228-2

**Published:** 2021-07-01

**Authors:** Alysha Cooper, Julie Horrocks, Sarah Goodday, Charles Keown-Stoneman, Anne Duffy

**Affiliations:** 1 Department of Mathematics and Statistics, University of Guelph, Guelph, ON Canada; 2 Department of Psychiatry, University of Oxford, Oxford, UK; 3 4YouandMe, Seattle, USA; 4 Dalla Lana School of Public Health, University of Toronto, Toronto, ON Canada; 5 Department of Psychiatry, Queen’s University, Kingston, ON Canada

**Keywords:** Bipolar spectrum, Major mood disorder, Prediction, Neural network, Longitudinal, High-risk offspring

## Abstract

**Background:**

Bipolar disorder onset peaks over early adulthood and confirmed family history is a robust risk factor. However, penetrance within families varies and most children of bipolar parents will not develop the illness. Individualized risk prediction would be helpful for identifying those young people most at risk and to inform targeted intervention. Using prospectively collected data from the Canadian Flourish High-risk Offspring cohort study available in routine practice, we explored the use of a neural network, known as the Partial Logistic Artificial Neural Network (PLANN) to predict the time to diagnosis of major mood disorders in 1, 3 and 5-year intervals.

**Results:**

Overall, for predictive performance, PLANN outperformed the more traditional discrete survival model for 3-year and 5-year predictions. PLANN was better able to discriminate or rank individuals based on their risk of developing a major mood disorder, better able to predict the probability of developing a major mood disorder and better able to identify individuals who would be diagnosed in future time intervals. The average AUC achieved by PLANN for 5-year prediction was 0.74, which indicates good discrimination.

**Conclusions:**

This evaluation of PLANN is a useful step in the investigation of using neural networks as tools in the prediction of mood disorders in at-risk individuals and the potential that neural networks have in this field. Future research is needed to replicate these findings in a separate high-risk offspring sample.

**Supplementary Information:**

The online version contains supplementary material available at 10.1186/s40345-021-00228-2.

## Background

Bipolar disorder affects an estimated 2.5% of the population, with higher prevalence for spectrum conditions (Merikangas et al. 2007). The onset peaks in late adolescence and early adulthood (Manchia et al. 2008); however, delayed recognition and misdiagnosis remains a challenge. Untreated illness is associated with substantial morbidity and mortality early in the course (Kessing et al. 2015), and therefore timely and accurate diagnosis is critical to facilitate prompt treatment.

Bipolar disorder runs in families, and therefore the children of bipolar parents are an identifiable high-risk group ideally suited for risk prediction studies (Duffy et al. 2017). Family studies have shown that bipolar-related mood disorders segregating in families includes major depressive disorder, bipolar I, II and schizoaffective bipolar disorder (Smoller and Finn 2003; McMahon et al. 2010; Craddock and Sklar 2013). The penetrance and spectrum of phenotypes vary between families and according to the subtype of bipolar illness. Longitudinal prospective studies of high-risk offspring have provided strong evidence that the illness often debuts with depressive episodes years before any hypomanic or manic episodes (Duffy et al. 2019).

While key risk factors for the development of bipolar disorder have been identified such as parental age of onset and clinical course, early adversity, and antecedent clinically significant symptoms (Preisig et al. 2016; Duffy et al. 2016), translatable risk prediction tools for clinicians do not exist or are in the early stages of development. For example, to our knowledge there has only been one published individualized risk calculator based on data from the BIOS longitudinal study of children of bipolar parents (Hafeman et al. 2017), which is yet to be replicated on an independent sample of familial at-risk offspring.

For studying events that occur over time, specialized modelling techniques that accommodate censoring are required (Allison 2010; Collett 2015). At the time the data analysis is conducted, not all individuals will necessarily have experienced the event of interest (bipolar-related mood disorders), and methods of survival analysis have been developed to accommodate these “censored” individuals, without introducing bias. In a longitudinal study, information on exposures and symptoms that might affect the risk of experiencing the outcome is collected repeatedly over time. Specialized techniques are necessary to include these repeatedly measured time-varying predictors in the analysis, while ensuring that only those exposures which occur before the outcome event are counted as possibly affecting the outcome. These techniques allow us to include the most up-to-date information collected on exposures, study the effect of symptoms that may not be present at baseline but develop during the course of follow-up, and ensure that the timing of the exposures in relation to the event of interest is taken into account. The latter is important as risk may be increased immediately after the exposure onset compared to later in time. Also, these techniques allow us to include numerical covariates that change over time, such as cumulative number of exposures at each timepoint. The Cox model (Cox 1972) and the discrete survival model (Efron 1988) are two such techniques from statistical survival analysis.

Recently, the use of neural networks has become increasingly popular in research for risk prediction (Charati et al. 2018; Wunnava et al. 2019; Li et al. 2017; LaFaro et al. 2015). An advantage of neural networks is that they do not rely on assumptions such as the distribution of the response variable or proportional hazards. Furthermore, they automatically accommodate non-linear relationships between the response and exposure variables (Biganzoli et al. 1998; Bourquin et al. 1997). Thus, rather than the researcher having to postulate a complicated, possibly non-linear model, the neural network can just “learn” the relationship with minimal direction from the researcher.

The purpose of this article is to explore the use of a neural network known as Partial Logistic Artificial Neural Network (PLANN) (Biganzoli et al. 1998), to predict the time to diagnosis of bipolar-related major mood disorders in the offspring of parents with confirmed bipolar disorder. For context, we compare PLANN to the more traditional discrete survival model. Both approaches accommodate censoring and time-varying predictors. The models are compared using several measures that assess the accuracy of the predictions. The prediction of which offspring are at greater risk of major mood disorder over time is important for clinical researchers, as it may allow for more proactive monitoring and prevention.

## Methods

### Study design

For this study, we used the data collected as part of the ongoing Canadian longitudinal high-risk offspring study described in detail elsewhere (Duffy et al.  2014, 2019). The study design is a dynamic, prospective cohort study. Briefly, original study families were identified through parents with bipolar I disorder confirmed by SADS-L interview and blind consensus review of all available clinical information. Subsequently, pedigrees were expanded and included first degree relatives of the original probands, who themselves were affected with bipolar-related major mood disorders (bipolar I, II, recurrent major depression). Agreeable offspring ages 5–25 years were enrolled and completed face to face research interviews following KSADS-PL format and study measures at baseline and then followed-up prospectively on average annually. This study has been reviewed for ethical compliance by the Ottawa Independent Research Ethics Board and the Queen’s University Health Science Research Ethics Board.

### Characteristics of participants and variables

In this analysis, we included 304 high-risk offspring from the Canadian high-risk cohort. The final data analysis was based on 292 high-risk individuals with no missing data for the predictors of interest. The outcome was defined as a DSM-IV diagnosis of bipolar-related major mood disorder including: bipolar disorder (Bipolar I, II, NOS), major depressive disorder and/or schizoaffective disorder based on semi-structured KSADS-PL format interviews and blind consensus review based on all available clinical and research material. Participants who did not have a diagnosis of major mood disorder before their last follow-up visit were considered censored at their last visit.

We limited variables in the model to those that would be relevant and routinely collected by clinicians in an office setting. Time-fixed predictors included sex at birth, parental response to lithium prophylaxis, parental age of onset of bipolar diagnosis, and childhood physical/sexual abuse. Time-varying predictors included the absence or presence at a given age of antecedent clinically significant symptoms and non-mood disorders (occurring prior to the outcome). In addition, the cumulative number of antecedent major and minor mood episodes at each age (occurring prior to the outcome) were included as time-varying predictors. Clinically significant activation (hypomanic), depressive, anxiety symptoms falling short of diagnostic criteria, as well as substance misuse and sleep problems were quantified based on clinical research interview and previously published consensus criteria (Duffy et al. 2019) (see Additional file 1). Childhood physical and sexual abuse was determined in offspring 13 years of age and older using the Childhood experiences of care and Abuse Scale (Bifulco et al. 2005), Note that only information up until time of diagnosis of major mood disorder or last follow-up visit was used in constructing the time-varying predictors. The time scale for both models was age of participant. In all analyses, covariates were lagged by one time interval in order to reduce the risk of reverse-causality and to enable predictions in the future time interval.

### Statistical analysis

PLANN and the discrete survival model are similar in many respects. Both approaches can accommodate time-fixed and time-varying predictors and use the same data setup (see Additional file 1). Both approaches predict the probability that an individual will experience the outcome within a given time frame, conditional on the individual not yet having experienced the outcome. However, the internal calculations performed to make the predictions are different (see Additional file 1), resulting in different values for the predicted probabilities. The discrete survival model assumes that odds are proportional at each time point and that the relationship between the hazard (risk) of the event and covariates is linear on the logistic scale, whereas PLANN makes no such assumptions (Allison 2010; Biganzoli et al. 1998). Details of the two models are included in the Additional file 1.

Models were evaluated using several assessment measures. The time-dependent c-index (Antolini et al. 2005) was used to quantify how well the model can rank individuals on their time to developing the outcome. The area under the receiver operating curve (AUC) is often used as a measure of the model’s ability to discriminate low and high-risk individuals (Zhou et al. 2011). Both the c-index and AUC range between 0 and 1, with 1 being best and a value greater than 0.5 being better than chance. The Brier score (Graf et al. 1999) measures the difference between the predicted probability of the event not occurring by a given follow-up time and the observed status of the individual at that time. The Brier score ranges between 0 and 1, with lower scores being better, 0 indicating perfect calibration and 0.25 indicating a non-informative model that is no better than chance. Common measures of prediction performance were used including accuracy, sensitivity, specificity, and positive predictive value. We used tenfold stratified cross-validation (see Additional file 1) to evaluate the predictive performance of the two models over 1-year, 3-year, and 5-year time intervals. For percentages, 95% confidence intervals were calculated as 100 × (p ± 1.96 × sqrt(p × (1−p)/n)) where p is the observed proportion and n is the sample size. For means m, 95% confidence intervals were calculated as m ± 1.96 × (sd/sqrt(n)) where sd is the standard deviation.

## Results

Table 1 presents the observed percent or means (and 95% confidence intervals) of the predictor variables included in the analyses. About 41% of participants were male. The mean age (at the time of the outcome event or censoring) was 21 years, and the mean age of parental onset was about 25 years. Childhood abuse was reported by 10% of offspring, while 38% were missing information on this variable as result of not being age appropriate for the measure or not yet completing the measure on next research visit. For the time-varying predictors, reported percentages/means are for disorders/episodes experienced before the outcome event or censoring. Over 41% of individuals had at least one clinically significant sub-threshold symptom presentation, with the most prevalent being subthreshold anxiety at 15.4%. The most prevalent disorder was anxiety disorder at close to 30%.

**Table 1:** Percent observed or mean of all variables included as covariates in the models and 95% confidence intervals for the percent or mean, for full sample (n = 292) and individuals with the outcome (n = 112)

Variable	Full sample (n = 292)		Individuals with outcome (n = 112)	
	n (%) or mean	[95% CI]	n (%) or mean	[95% CI]
Time-fixed				
Sex (male)	121 (41.4)	[35.8, 47.1]	35 (31.3)	[22.6, 39.9]
Age (at outcome or censoring)	21.0	[20.2, 21.9]	19.6	[18.6, 20.6]
Parental lithium response	129 (44.2)	[38.5, 49.9]	47 (42.0)	[32.8, 51.1]
Parental onset age	25.4	[24.4, 26.5]	25.5	[23.7, 27.4]
Physical/sexual abuse				
Yes	30 (10.3)	[6.1, 13.8]	14 (12.5)	[6.3, 18.7]
Missing^a^	111 (38.0)	[32.4, 43.6]	32 (28.6)	[20.2, 37.0]
Time-Varying				
Disorders				
Subthreshold^b^ activation	29 (9.9)	[6.5, 13.4]	19 (17.0)	[10.0, 23.9]
Subthreshold depression	31 (10.6)	[7.1, 14.2]	17 (15.2)	[8.5, 21.6]
Subthreshold sleep	9 (3.1)	[1.1, 5.1]	2 (1.8)	[0.0, 4.2]
Subthreshold substance use	36 (12.3)	[8.6, 16.1]	13 (11.6)	[5.6, 17.6]
Subthreshold anxiety	45 (15.4)	[11.3, 19.6]	17 (15.2)	[8.5, 21.9]
Substance use	51 (17.5)	[13.1, 21.8]	29 (25.9)	[17.7, 34.0]
Sleep	54 (18.5)	[14.0, 23.0]	18 (16.1)	[9.2, 22.9]
Anxiety	87 (29.8)	[24.5, 35.0]	39 (34.8)	[26.0, 43.7]
Neurodevelopmental	33 (11.3)	[7.7, 14.9]	12 (10.7)	[5.0, 16.5]
Number of episodes				
Major mood	0.2	[0.1, 0.3]	0.2	0.1, 0.3]
Minor mood	0.2	[0.1, 0.3]	0.1	[0.0, 0.2]

^a^Missing refers to having not completed the measure either due to not being age appropriate to complete or not yet completing the measure; ^b^see Additional file 1 for definitions of subthreshold conditions

Out of the 292 individuals included in the analysis, 112 (38.4%) developed a major mood disorder by their last follow-up visit, while 180 (61.6%) did not and were censored. As shown in the right-hand column of Table 1, proportionately fewer males experienced the outcome than were present in the full sample (31% versus 41%), and those who experienced the outcome were slightly younger on average (19.6 versus 21.0 years). More of those diagnosed with a major mood disorder experienced substance use, anxiety, and subthreshold activation and depression than in the full at-risk offspring sample.

### Model comparisons

Table 2 compares PLANN and the discrete survival model in terms of the assessment measures, averaged across time intervals and using tenfold cross-validation, for 1-, 3- and 5-year predictions. Recall that for the Brier score, lower is better, while for the c-index and AUC, higher is better. PLANN outperformed the discrete survival model on the Brier score which measures the difference between observed and predicted values. The c-index indicates how well the model ranks individuals in terms of their event times. PLANN outperformed the discrete survival model at all three interval lengths. On mean AUC, PLANN outperformed the discrete survival model in 3-year and 5-year predictions, but the opposite is true in 1-year predictions. PLANN does better at 5-year predictions than at 1- or 3-year predictions according to mean AUC. The 5-year mean AUC for PLANN was 0.74.

**Table 2:** Comparison of the evaluation metrics for PLANN and the discrete survival model with 1-year, 3-year and 5-year predictions

	One-year		Three-year		Five-year	
	PLANN	survival	PLANN	survival	PLANN	survival
Mean Brier Score^a^	0.177	0.180	0.182	0.188	0.179	0.190
C-index	0.633	0.556	0.549	0.513	0.590	0.520
Mean AUC(SD)^b^	0.633 (0.170)	0.740 (0.144)	0.708 (0.123)	0.655 (0.124)	0.745 (0.076)	0.669 (0.063)

^a^The mean Brier score is the average Brier score across 15, 20, and 25 years

^b^The mean AUC is the average AUC across the time intervals measured

Table 3 shows mean accuracy, specificity, sensitivity, and positive predictive value (PPV), for 1-year, 3-year, and 5-year predictions for the two models, where the mean is taken across time intervals and tenfold cross-validation was used (see Additional file 1 for more details). For 1-year prediction, PLANN outperformed the discrete survival model with higher sensitivity, specificity, and PPV when the optimal threshold was used. However, for 3- and 5-year predictions, PLANN and the discrete survival model gave similar results for the optimal thresholds. A trade-off between specificity and sensitivity was demonstrated for both models across all prediction intervals when examining performance across different thresholds. With lower thresholds, sensitivity is high and specificity is low, whereas the opposite was true for higher thresholds. Optimal thresholds achieve a balance between sensitivity and specificity.

**Table 3:** Mean accuracy, specificity, sensitivity, and positive predictive value (PPV) across time intervals and 10 CV-folds for 1-year, 3-year, and 5-year predictions for PLANN and the discrete survival model

Model	Threshold	Accuracy	Specificity	Sensitivity	PPV
One year					
PLANN^a^					
	0.05	0.751	0.762	0.272	0.064
	0.10	0.948	0.986	0.019	0.051
	0.15	–	–	–	–
	0.20	–	–	–	–
	Optimal^b^	0.681	0.683	0.488	0.081
Survival					
	0.05	0.772	0.783	0.360	0.073
	0.10	0.895	0.924	0.186	0.100
	0.15	0.927	0.960	0.128	0.133
	0.20	0.940	0.976	0.083	0.160
	Optimal^b^	0.556	0.560	0.383	0.043
Three year					
PLANN					
	0.05	0.269	0.186	0.822	0.107
	0.10	0.588	0.583	0.443	0.111
	0.15	0.762	0.808	0.257	0.167
	0.20	0.818	0.899	0.096	0.143
	Optimal^b^	0.591	0.591	0.506	0.134
Survival					
	0.05	0.270	0.181	0.791	0.101
	0.10	0.533	0.513	0.483	0.111
	0.15	0.760	0.813	0.218	0.125
	0.20	0.841	0.919	0.128	0.134
	Optimal^b^	0.572	0.582	0.474	0.130
Five year					
PLANN					
	0.05	0.222	0.070	0.934	0.162
	0.10	0.425	0.335	0.709	0.169
	0.15	0.582	0.546	0.565	0.210
	0.20	0.687	0.722	0.360	0.213
	0.607	0.617	0.577	0.230	0.607
Survival					
	0.05	0.264	0.116	0.893	0.161
	0.10	0.410	0.300	0.662	0.143
	0.15	0.548	0.511	0.508	0.160
	0.20	0.709	0.738	0.420	0.238
	Optimal^b^	0.601	0.615	0.515	0.224

^a^One-year predictions made by PLANN were all below 0.15 and therefore, thresholds of 0.15 or greater could not be evaluated for accuracy metrics

^b^The Optimal rows present the average accuracy metrics across time intervals when the optimal threshold of each time interval is used (see Additional file 1)

**Fig. 1:**
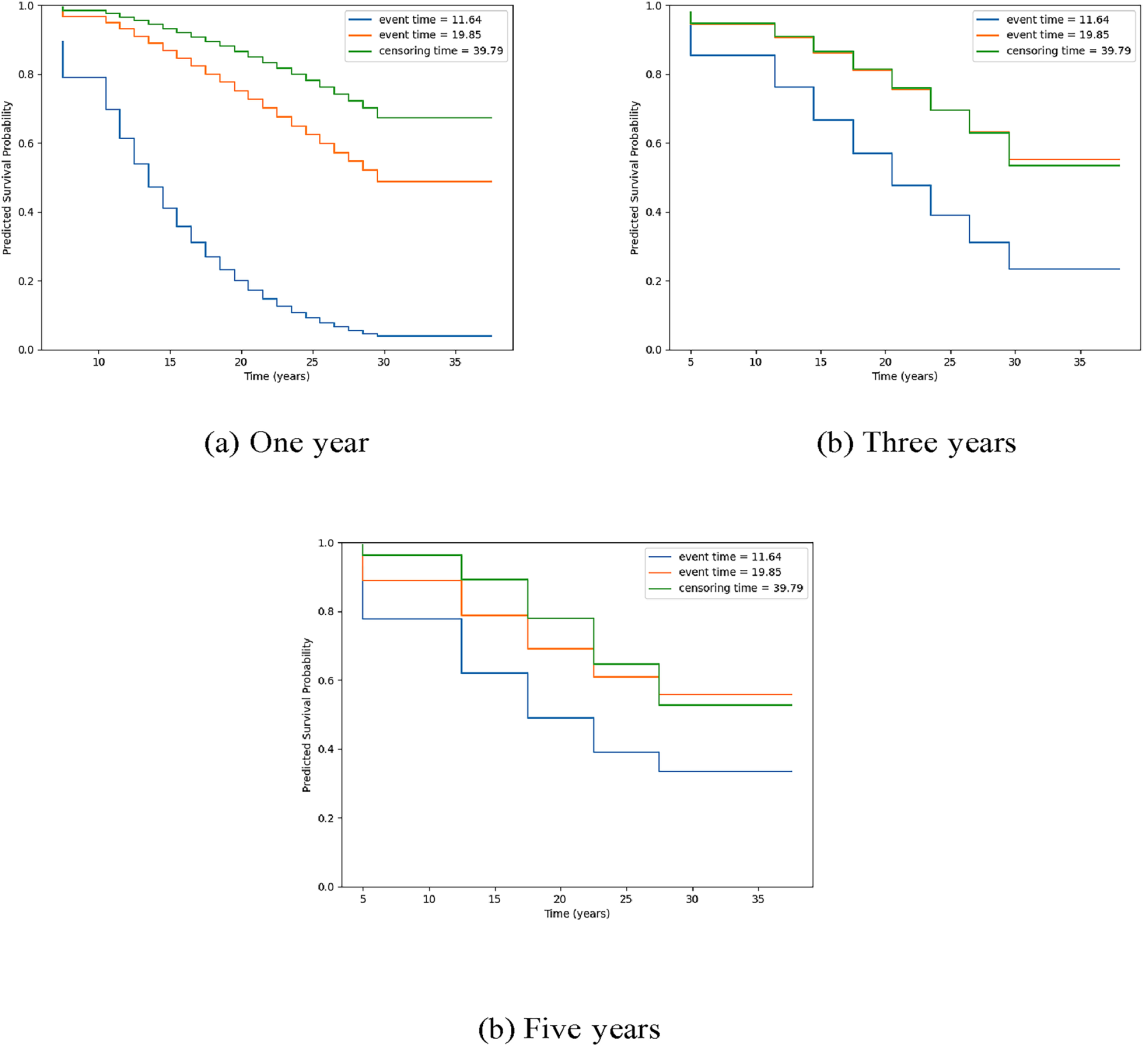
Predicted survival curves for ‘earlier-onset’, ‘mid-onset’ and ‘no onset’ individuals in test set of single fold for PLANN with (**a**) 1 year, **b** 3 year and (**c**) 5-year predictions. Legend: blue line indicates high-risk offspring with earlier-onset, orange line indicates mid-onset and green line indicates no-onset of bipolar-related major mood disorder over observation time

Finally, it was of interest to assess whether the models could distinguish between three individuals in the test set selected based on their observed diagnosis and censoring time. The individual in the test set with the earliest diagnosis time was considered the ‘earlier-onset’ individual, the individual with the median diagnosis time was considered the ‘mid-onset’ individual and the individual with the highest censoring time (longest survival time) was the ‘no onset’ individual. The ‘earlier-onset’ individual experienced a major mood disorder at 11.64 years, the ‘mid-onset’ individual was diagnosed at 19.85 years and the ‘no onset’ individual was censored at 39.79 years. For these three individuals, the predicted survival curves were plotted for 1 year, 3 year and 5-year predictions made by PLANN (Fig. 1). PLANN predicted that the ‘earlier-onset’ individual had the lowest survival probability over time.

**Fig. 2:**
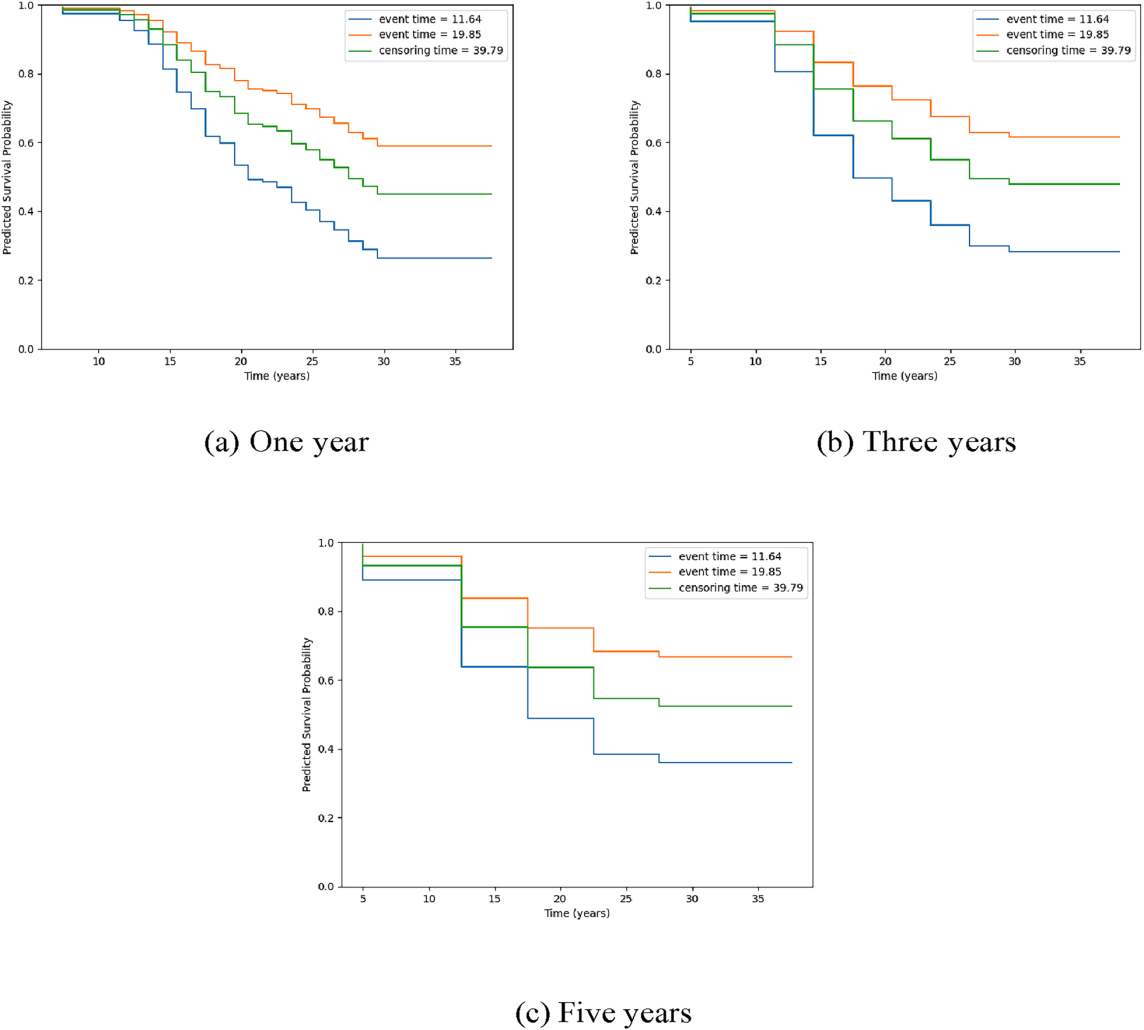
Predicted survival curves for **‘**earlier-onset’, ‘mid-onset’ and ‘no onset’ individuals in test set of single fold for the discrete survival model with (**a**) 1 year, **b** 3 year and (**c**) 5 year predictions. Legend: blue line indicates high-risk offspring with earlier-onset, orange line indicates mid-onset and green line indicates no-onset of bipolar-related major mood disorder over observation time

When making 1-year predictions of major mood disorder, PLANN could predict that the ‘mid-onset’ individual had a lower survival probability than the ‘no onset’ individual. However, PLANN had more difficulty distinguishing between the ‘mid-onset’ and ‘no onset’ individuals when three and five-year predictions were made. For these individuals, the predicted survival curves were additionally plotted for the discrete survival model with one-year predictions, three-year predictions and five-year predictions. As seen in Fig. 2, the discrete survival model predicted that the ‘earlier-onset’ individual had a higher probability of diagnosis (i.e. lower survival probability) over time compared to the ‘mid-onset’ and ‘no onset’ individuals. However, for 1-year, 3-year and 5-year predictions, the discrete survival model predicted that the ‘mid-onset’ individual had a higher probability of not being diagnosed than the ‘no onset’ individual. These findings further demonstrate that PLANN outperforms the discrete survival model in discrimination of higher-risk versus lower-risk offspring.

## Discussion

In this study we explored the potential utility of using Partial Logistic Artificial Neural Network (PLANN), an extension of discrete survival analysis, to predict time to diagnosis of major mood disorder at 1, 3 and 5 years into the future in a well-characterized prospectively followed cohort of high-risk individuals identified based on a parent with bipolar disorder. We limited fixed and time-varying covariates in the model to data that would be routinely collected and available in clinical practice (i.e., sex, age, childhood abuse, subthreshold antecedent clinically significant symptoms and lifetime antecedent non-mood diagnoses). We included major depressive disorder as part of the bipolar-related major mood disorders given (i) major depression is considered part of the bipolar spectrum in genetic studies (McMahon et al. 2010; McGuffin et al. 2003; Coleman et al. 2020) and (ii) bipolar disorder typically debuts as major depression in high-risk offspring of bipolar parents (Duffy et al. 2017; Mesman et al. 2013).

PLANN was compared to the more traditional discrete survival model to assess whether the use of a neural network provides any benefit over a traditional statistical modeling approach. While PLANN and the logistic model have common advantages, such as enabling the incorporation of time-varying covariates due to the use of discrete time intervals, both models also have distinct advantages over one another. The logistic model allows for the interpretation of the effect of covariates on the discrete hazard and the evaluation of whether or not the covariates have a significant effect on the discrete hazard, although this was not pursued here. On the other hand, PLANN makes fewer assumptions about the relationship between the outcome and covariates and has the ability to automatically detect non-linear relationships in the data. The latter point is important, as for example, mood instability has been found to follow non-linear patterns (Bonsall et al. 2012). A drawback of PLANN, and machine learning models in general, is that they are computationally intensive, requiring high-performance computer clusters and many days of computation, whereas discrete survival models take only a few seconds to fit.

Overall, for predictive performance, PLANN outperformed the logistic model for 3-year and 5-year predictions. PLANN was better able to discriminate or rank individuals based on their risk of developing major mood disorder (i.e., higher time-dependent c-indices) and better able to predict the probability of developing major mood disorder (i.e., lower Brier scores). The results were mixed for 1-year predictions, with the discrete survival model outperforming PLANN for AUC. For five-year predictions, the average AUC from PLANN was 0.74, which indicates that the model shows good discrimination between high and low risk individuals. This value is comparable to (Hafeman et al. 2017) who achieved an AUC of 0.76 for 5-year predictions.

Prediction was superior in the 3 and 5-year models compared to the 1-year models, as evidenced by higher AUC, sensitivity and positive predictive values. This is not surprising, as relatively few events occurred in specific 1-year intervals, compared to three or 5 year intervals (see Additional file 1: Tables S2 and S3). Also, it is intuitively more difficult to predict that an event will occur in a 1-year interval, than to say it will happen sometime over the next 5 years.

Individualized risk prediction has important implications for research and clinical practice. By identifying from among individuals at familial risk those most likely to develop illness, an ultra-high risk group can be identified that can inform research into the determinants of illness onset and prevention. Moreover, this ultra-high risk group would be suitable and would likely benefit from prospective surveillance and low-risk intervention and psychoeducation targeting sleep hygiene, healthy coping and stress reduction, healthy lifestyle and diet and avoidance of alcohol misuse and drug use. Our risk prediction approach of using PLANN to predict onset of bipolar-related major mood disorder differs from other published risk calculators such as (Hafeman et al. 2017), which used a “baseline re-setting” Cox proportional hazards model. Both the Cox model and PLANN allow the inclusion of covariates measured at baseline and at follow-up visits and neither method requires an assumption about the distribution of the outcome variable. However, unlike the Cox model, PLANN does not require a proportional hazards assumption. In addition, we only included model variables in PLANN that would be available in routine practice.

## Strengths and limitations

Strengths include the carefully assessed parental diagnoses based on longitudinal clinical observations confirming the risk status in the offspring, the measurement of diagnosis in high-risk offspring through semi-structured research clinical assessments and blind consensus reviews. However, the following limitations relevant to this analysis are worth noting. The sample size is small, particularly for neural networks, which typically require sample sizes in the thousands; it is notoriously difficult to make predictions in medicine, due to lack of relevant variables (Lawless 2010) and thus additional breadth of data (e.g., genetic data, behavioural data) may improve predictions.

## Conclusion

This evaluation of PLANN is a useful step in the investigation of using neural networks as tools in the prediction of diagnosis of mood disorders for at-risk individuals and demonstrated the potential that neural networks have in this field. PLANN performed better than the traditional discrete time survival model in predicting the development of major mood disorders in high-risk individuals. Future research replicating these approaches in different samples with the inclusion of additional data will help inform the further utility of risk prediction models to aid in research and clinical decision making in individuals at familial risk of developing bipolar-related mood disorders.

## Supplementary Information

**Additional file 1: Table S1.** Example of discrete survival data for hypothetical scenario with sex and blood pressure as covariates. **Figure S1.** Feed-forward neural network with one hidden layer. **Table S2.** PLANN one-, three-, five-year prediction assessments showing optimal threshold, accuracy, specificity (spec), sensitivity (sens), positive predictive value (PPV), total number of individuals in the risk set (n) and total number of events observed within each time interval, determined using tenfold cross-validation. **Table S3.** Discrete survival model one-, three-, five-year prediction assessments showing optimal threshold, accuracy, specificity (spec), sensitivity (sens), positive predictive value (PPV), total number of individuals in the risk set(n) and total number of events observed within each time interval, determined using tenfold cross-validation. **Table S4.** Number of subthreshold diagnoses for individuals in the full sample, individuals who experienced the major mood disorder outcome (diagnosis of BP, MDD and/or schizoaffective disorder), and individuals who did not experience the outcome.

## Data Availability

This is an ongoing study and requests for access to analyze de-identified study data may be made to the principal investigator Dr Anne Duffy as per ethically reviewed study protocol.

## Abbreviations

BD: Bipolar Disorder
CECA: Childhood experience of care and abuse
DSM: Diagnostic and Statistical Manual for Mental Disorders
K-SADS–PL: Kiddie Schedule for Affective Disorders–Present and Lifetime
PLANN: Partial Logistic Artificial Neural Network
SADS-L: Schedule for Affective Disorders-Lifetime
