# Supplementary material for: Predicting the risk and timing of major mood disorder in offspring of bipolar parents: exploring the utility of a neural network approach
Source: Int J Bipolar Disord. 2021 Jul 1;9:22. doi: 10.1186/s40345-021-00228-2 (PMC8245610; doi:10.1186/s40345-021-00228-2)
Supplement: Supplementary file 1 — Additional file 1: Table S1. Example of discrete survival data for hypothetical scenario with sex and blood pressure as covariates. Figure S1. Feed-forward neural network with one hidden layer. Table S2. PLANN one-, three-, five-year prediction assessments showing optimal threshold, accuracy, specificity (spec), sensitivity (sens), positive predictive value (PPV), total number of individuals in the risk set (n) and total number of events observed within each time interval, determined using tenfold cross-validation. Table S3. Discrete survival model one-, three-, five-year prediction assessments showing optimal threshold, accuracy, specificity (spec), sensitivity (sens), positive predictive value (PPV), total number of individuals in the risk set(n) and total number of events observed within each time interval, determined using tenfold cross-validation. Table S4. Number of subthreshold diagnoses for individuals in the full sample, individuals who experienced the major mood disorder outcome (diagnosis of BP, MDD and/or schizoaffective disorder), and individuals who did not experience the outcome. [file 40345_2021_228_MOESM1_ESM.docx]

**Additional file 1**

*Data Setup for both models*

For both PLANN and discrete survival analysis, follow-up time is divided into K discrete intervals:

(*t*_0_*, t*_1_]*,* (*t*_1_*, t*_2_]*, . . . ,* (*t_k−_*_1_*, t_k_*]*, . . . ,* (*t_K−_*_1_*, t_K_*].

Individuals contribute data to the analysis only in intervals prior to the outcome event or censoring. The observed response variable, δ_ik_, is binary. It equals 1 if individual i experienced the event in the k^th^ time interval, (t_k−1_, t_k_] and 0 otherwise. In Supplementary Table 1 we show hypothetical data for two individuals with covariates sex (time-fixed) and blood pressure (time-varying). Individual 1 is censored in the 3^rd^ interval and so contributes 3 intervals to the analysis. Individual 2 has an event in the 4^th^ interval and so contributes 4 intervals to the analysis. Note that for individuals who are censored, δ_ik_ =0 for all contributed time intervals. For individuals who experience the event in the j^th^ time interval, δ_ij_ = 1 and for all prior intervals, δ_ik_ =0.

**Table S1**: Example of discrete survival data for hypothetical scenario with sex and blood pressure as covariates.

| **Patient ID** | **Sex** | **Blood Pressure** | **Time**  **Interval** |  |  | **Event Status δ_ik_** |  |
| --- | --- | --- | --- | --- | --- | --- | --- |
| 1 | 1 | 120/80 | 1 | |  | 0 | |
| 1 | 1 | 125/82 | 2 | |  | 0 | |
| 1 | 1 | 122/80 | 3 | |  | 0 | |
| 2 | 0 | 110/60 | 1 | |  | 0 | |
| 2 | 0 | 110/58 | 2 | |  | 0 | |
| 2 | 0 | 108/60 | 3 | |  | 0 | |
| 2 | 0 | 105/55 | 4 | |  | 1 | |

For the analyses reported in this article, follow-up time was divided into one-, three- or five-year intervals. Very few diagnoses occurred prior to ten years of age or after 30 years of age. Therefore, for each analysis, the time intervals were longer at the beginning and end of the follow-up period to ensure that each time interval contained at least one event. The first time interval for all analyses was (0-10] years of age and the final time interval was (30-45] years of age or (31-45] years in the case of the three year time intervals.

For the time-varying covariates, if multiple measures were taken for a given covariate within a single time interval, the most recent of the measures was used. In addition, the time-varying covariates were lagged by one-time interval in order to reduce the risk of reverse-causality and to make predictions in the future time interval.

*Discrete Survival Model*

The discrete survival model was introduced by Cox^1^ in 1972 and further developed by Efron^2^.

It is applicable more generally to any outcome of interest, not just death/survival, and can accommodate both time-fixed and time-dependent covariates. The discrete survival model specifies a particular relationship between the discrete hazard and covariates. The discrete hazard, *h_ik_*, is defined as the conditional probability that individual *i* experiences the event of interest in the *k^th^* time interval, (*t_k−_*_1_*, t_k_*], given that they did not experience the event beforehand. The discrete survival model uses the logistic function to define the relationship between the discrete hazard and the covariates:

*h_ik_* = [1 + exp(*−*[*α_k_* + *β*_1_*x_ik_*_1_ + *β*_2_*x_ik_*_2_ + *...* + *β_p_x_ikp_*])]*^−^*^1^*,* (1)

where *α_k_* is the intercept for the *k^th^* time interval, *x_ik_*_1_, *x_ik_*_2_, *. . .*, *x_ikp_* are the values of the covariates for individual *i* in interval *k* and *β*_1_*, β*_2_*, . . . , β_p_* are the corresponding regression parameters which will be estimated. Note that this model specification ensures that odds *h_ik_*/(1- *h_ik_)* in any time interval are proportional, and that the relationship between hazard and covariates is linear on the logistic scale^3^. The parameters are estimated through maximization of the log likelihood function.

The survival function, *S_ik_*, gives the probability that the event occurs after time *t_k_* for individual *i*. It is easily calculated as:

*S_ik_* = $\prod_{r=1}^{k} h_{ir}$*.* (2)

Note that *S_ik_* decreases over time. A model-free estimate of this function is the familiar Kaplan-Meier estimator^4^.

*Neural Networks*

The goal of neural networks is to learn the relationship between a set of predictors and the response. The building blocks of neural networks are known as nodes, which are organized into layers and connected to one another through weights. Feed-forward neural networks have an input layer, one or more hidden layer(s) and an output layer. The information is distributed through the neural network in one direction, beginning at the input layer and finishing at the output layer^5^.

Figure S1 shows the process of prediction for a single individual using a feed-forward neural network with a single hidden layer. The nodes of the neural network are represented by circles and each layer is represented by a vertical stack of nodes. The weights of the network are represented by the lines and the arrows indicate the direction in which the information is passed through the network. The covariates for individual *i* are denoted as *x_i_*_1_*, x_i_*_2_*, ..., x_ip_*, the hidden layer features are denoted as *z_i_*_1_*, z_i_*_2_*, ..., z_iH_* , and the predictions are denoted as *y*ˆ*_i_*_1_*, y*ˆ*_i_*_2_*, . . . , y*ˆ*_iK_*. The nodes in the hidden layer and output layer are determined by taking a weighted sum of the nodes in the previous layer and applying a function, known as the *activation function*, *φ*, to the weighted sum:

*z_ih_* = *φ_h_(*$\sum_{j=0}^{p} w_{jh}x_{ij}$*)* (3)

*y*ˆ*_i_* = *φ_o_(*$\sum_{h=0}^{H} \omega_{h}z_{ih}$*)* (4)

where the *w*’s and *ω*’s are weights to be estimated. Note that *x_i_*_1_ = 1 and *z_i_*_1_ = 1 so that *w_h_*_1_ and *ω*_1_ are intercepts.

Learning the relationship between the covariates and the target response is known as training the network. To train the network, the weights of the network are optimized to minimize a loss function which compares the prediction to the target response for individuals in the training set. The loss function selected is dependent upon the problem which the neural network is solving^5^.

Prior to training the neural network, certain quantities called hyper-parameters must be determined. This part of the process can be the most time consuming, as it requires the neural network to be fit to the data numerous times.

**Figure S1:** Feed-forward neural network with one hidden layer


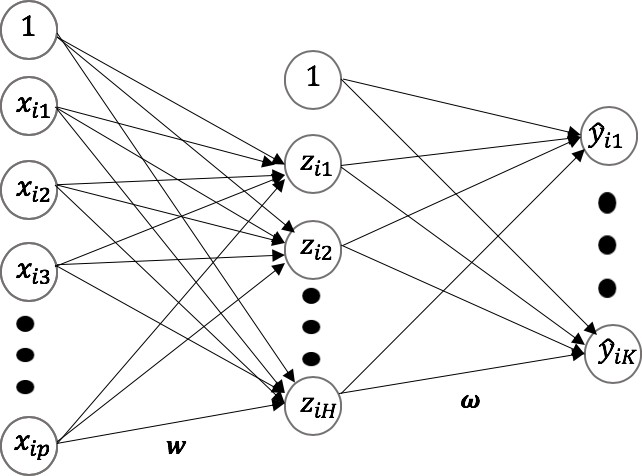


***PLANN***

Partial Logistic Artificial Neural Network (PLANN) is a feed-forward neural network with one hidden layer that was developed by Biganzoli et al.^6^. Like the discrete survival model, the time axis is divided into K intervals, the response in each interval is 1 if the event occurred and 0 otherwise. PLANN has a single output which is the predicted discrete hazard for a given time interval. The inputs are therefore the time interval of interest and the values of the covariates in the given time interval. The loss function used to estimate the weights in PLANN is equivalent to the log-likelihood of the discrete survival model.

For PLANN, the activation functions are logistic.so that equations (3) and (4) above become

z_ikh_ = [1 + exp(*−*[*w*_1_*x_ik_*_1_ + *w*_2_*x_ik_*_2_ + *...* + *w_p_x_ikp_*])]*^−^*^1^ (5),

and

h_ik_ = [1 + exp(*−*[$\omega_{1}$*z_ik_*_1_ + $\omega_{2}$*z_ik_*_2_ + *...* + $\omega_{H}$*z_ikH_*])]*^−^*^1^ (6),

respectively, for the k^th^ time interval. The hidden layer is what makes PLANN different from discrete survival, by relaxing the direct relationship between hazard h_ik_ and covariates (x_ikj_) shown in (1).

Before training the model, we selected values for the following hyper-parameters: learning rate and momentum (used in minimizing the loss function)^7^ and the ridge regularization parameter (which determines to what degree the weights will be shrunk towards zero in order to avoid overfitting the model to the training set)^8^. We selected values of the hyper-parameters which maximized the time-dependent c-index.

**Model Assessment Measures**

***Time-Dependent C-Index***

The time-dependent c-index is a measure of discrimination performance, or in other words, how well the model can distinguish between high- and low-risk individuals, measured by calculating the proportion of pairs that are concordant^9^. In order for a pair to be concordant in a given time interval, the individual with the lower probability of the event occurring beyond the particular time, *S_ik_*, must have the lower observed event time. The time-dependent c-index finds the average concordance over the follow-up period.

***AUC***

Predictions of the conditional hazard in time interval *k* were made for individuals in the test set who were still at risk by the beginning of the time interval. The predictions of the conditional hazard were then compared to the true status of each individual to create the receiver operating characteristic (ROC) curve. The ROC curve plots sensitivity along the y-axis and the false positive rate (1-specificity) along the x-axis to present all possible specificity and sensitivity pairs across the range of possible thresholds of classification. The area under the ROC curve (AUC) is often used as a measure of accuracy for diagnostic models. An AUC of 1 indicates that the model has perfect accuracy, an AUC of 0 indicates that the model is perfectly inaccurate, and an AUC of 0.5 indicates that the model’s predictions perform no better than chance. Therefore, an AUC closer to 1 demonstrates that the model can distinguish between individuals with and without the condition better than chance^10^.

***Brier Score***

The Brier score is a measure of calibration performance, which measures how well the model predicts the observed response. The Brier score measures the difference between the predicted probability of the event not occurring by a given follow-up time and the observed survival status, *δ_ik_,* at that time. The Brier score ranges between 0 and 1 with 0 indicating perfect calibration and 0.25 indicating a non-informative model being no better than chance. The Brier score for censored data generalizes the Brier score for survival data^11^.

***Prediction***

We classified each individual at risk in interval *k* as positive if *h_ik_ > L_k_* where *L_k_* is a chosen threshold of risk. This was compared to the individual’s true status (positive or negative) in the next time interval (*k* + 1) in order to calculate sensitivity, specificity, positive predictive value and accuracy:

Sensitivity = $\frac{TP}{TP+FN}$,

Specificity = $\frac{TN}{FP+TN}$,

Positive predictive value (PPV) = $\frac{TP}{TP+FP}$ ,

and

Accuracy = $\frac{TP+TN}{TP+TN+FP+FN}$ .

*w*here *TP* is the number of true positives, *TN* is the number of true negatives, *FP* is the number of false positives and *FN* is the number of false negatives in the risk set for a given time interval^12^. We examined these prediction assessment measures with five thresholds to see how model performance varied across thresholds. The first four thresholds were arbitrary (i.e. 0.05, 0.10, 0.15, 0.20). The optimal threshold was selected to balance the sensitivity and specificity of the model through optimization of Youden’s J index^13^.

***Cross-validation***

Using the same data to both fit and assess the model can lead to overly-optimistic estimates of assessment. To alleviate this, 10-fold stratified cross validation was used for all assessments, in order to estimate how the results might generalize to an independent data set. The data set was divided into 10 equal sized subsets, each with the same proportion of individuals having the outcome. The model was trained on (fit to) the data 10 times, each time leaving out a different subset which was used as the test set, on which the evaluation measures were calculated. Finally, the evaluation measures were averaged across the 10 folds^14^.

Tables S2 and S3 show prediction assessment measures for PLANN and discrete survival respectively. For each test set in the cross-validation, we calculated accuracy, specificity, sensitivity and PPV in each time interval, using the optimal threshold for that interval. The average optimal threshold and results across the ten test sets are presented for each time interval. The average value across all time intervals is also shown in Tables S2 and S3 and is reported in Table 3 in the main text.

**Table S2.** PLANN one-, three-, five-year prediction assessments showing optimal threshold, accuracy, specificity (spec), sensitivity (sens), positive predictive value (PPV), total number of individuals in the risk set (n) and total number of events observed within each time interval, determined using 10-fold cross-validation.

| prediction interval width | time interval | threshold | accuracy | spec | sens | PPV | n | # events |
| --- | --- | --- | --- | --- | --- | --- | --- | --- |
| One-year |  |  |  |  |  |  |  |  |
|  | 10-11 | 0*.*153 | 0*.*932 | 0.935 | 0 | 0 | 277 | 1 |
|  | 11-12 | 0*.*220 | 0*.*993 | 0.996 | 0 | 0 | 271 | 1 |
|  | 12-13 | 0*.*029 | 0*.*786 | 0.792 | 0*.*333 | 0*.*017 | 261 | 3 |
|  | 13-14 | 0*.*025 | 0*.*643 | 0.649 | 0*.*250 | 0*.*010 | 252 | 4 |
|  | 14-15 | 0*.*021 | 0*.*455 | 0.457 | 0*.*400 | 0*.*015 | 240 | 6 |
|  | 15-16 | 0*.*047 | 0*.*618 | 0.625 | 0*.*500 | 0*.*169 | 229 | 12 |
|  | 16-17 | 0*.*047 | 0*.*776 | 0.779 | 0*.*813 | 0*.*237 | 209 | 11 |
|  | 17-18 | 0*.*048 | 0*.*769 | 0.764 | 0*.*600 | 0*.*084 | 190 | 8 |
|  | 18-19 | 0*.*036 | 0*.*577 | 0.569 | 0*.*571 | 0*.*072 | 174 | 11 |
|  | 19-20 | 0*.*027 | 0*.*365 | 0.361 | 0*.*625 | 0*.*029 | 160 | 5 |
|  | 20-21 | 0*.*074 | 0*.*761 | 0.760 | 0*.*857 | 0*.*145 | 148 | 10 |
|  | 21-22 | 0*.*083 | 0*.*767 | 0.788 | 0*.*400 | 0*.*075 | 132 | 6 |
|  | 22-23 | 0*.*170 | 0*.*560 | 0.571 | 0 | 0 | 115 | 1 |
|  | 23-24 | 0*.*079 | 0*.*646 | 0.652 | 0*.*500 | 0*.*019 | 108 | 2 |
|  | 24-25 | 0*.*056 | 0*.*490 | 0.475 | 0*.*500 | 0*.*067 | 101 | 7 |
|  | 25-26 | 0*.*075 | 0*.*651 | 0.650 | 0*.*667 | 0*.*067 | 90 | 3 |
|  | 26-27 | 0*.*080 | 0*.*634 | 0.618 | 0*.*750 | 0*.*088 | 79 | 5 |
|  | 27-28 | 0*.*126 | 0*.*849 | 0.856 | 0*.*750 | 0*.*281 | 68 | 4 |
|  | 28-29 | 0*.*087 | 0*.*544 | 0.532 | 0*.*750 | 0*.*106 | 59 | 5 |
|  | 29-30 | 0*.*118 | 0*.*793 | 0.827 | 0*.*500 | 0*.*143 | 45 | 2 |
|  | **mean** | **0.080** | **0.681** | **0.683** | **0.488** | **0.081** |  |  |
| Three-year |  |  |  |  |  |  |  |  |
|  | 10-13 | 0*.*054 | 0*.*669 | 0.674 | 0*.*400 | 0*.*018 | 277 | 5 |
|  | 13-16 | 0*.*056 | 0*.*464 | 0.469 | 0*.*350 | 0*.*050 | 252 | 22 |
|  | 16-19 | 0*.*080 | 0*.*549 | 0.535 | 0*.*635 | 0*.*170 | 209 | 30 |
|  | 19-22 | 0*.*114 | 0*.*668 | 0.710 | 0*.*333 | 0*.*139 | 160 | 21 |
|  | 22-25 | 0*.*113 | 0*.*461 | 0.464 | 0*.*357 | 0*.*065 | 115 | 10 |
|  | 25-28 | 0*.*155 | 0*.*649 | 0.630 | 0*.*688 | 0*.*243 | 90 | 12 |
|  | 28-31 | 0*.*204 | 0*.*673 | 0.653 | 0*.*778 | 0*.*250 | 59 | 8 |
|  | **mean** | **0.111** | **0.591** | **0.591** | **0.506** | **0.134** |  |  |
| Five-year |  |  |  |  |  |  |  |  |
|  | 10-15 | 0*.*087 | 0*.*570 | 0.572 | 0*.*604 | 0*.*061 | 277 | 15 |
|  | 15-20 | 0*.*133 | 0*.*546 | 0.532 | 0*.*662 | 0*.*264 | 229 | 47 |
|  | 20-25 | 0*.*220 | 0*.*642 | 0.680 | 0*.*460 | 0*.*245 | 148 | 26 |
|  | 25-30 | 0*.*257 | 0*.*669 | 0.686 | 0*.*583 | 0*.*350 | 90 | 19 |
|  | **mean** | **0.174** | **0.607** | **0.617** | **0.577** | **0.230** |  |  |

**Table S3.** Discrete survival model one-, three-, five-year prediction assessments showing optimal threshold, accuracy, specificity (spec), sensitivity (sens), positive predictive value (PPV), total number of individuals in the risk set(n) and total number of events observed within each time interval, determined using 10-fold cross-validation.

| prediction interval width | time interval | threshold | accuracy | spec | sens | PPV | n | # events |
| --- | --- | --- | --- | --- | --- | --- | --- | --- |
| One-year |  |  |  |  |  |  |  |  |
|  | 10-11 | 0*.*104 | 0*.*565 | 0.569 | 0 | 0 | 277 | 1 |
|  | 11-12 | 0*.*107 | 0*.*971 | 0.974 | 0 | 0 | 271 | 1 |
|  | 12-13 | 0*.*012 | 0*.*561 | 0.559 | 0*.*667 | 0*.*011 | 261 | 3 |
|  | 13-14 | 0*.*013 | 0*.*362 | 0.361 | 0*.*500 | 0*.*011 | 252 | 4 |
|  | 14-15 | 0*.*024 | 0*.*466 | 0.464 | 0*.*600 | 0*.*020 | 240 | 6 |
|  | 15-16 | 0*.*053 | 0*.*521 | 0.531 | 0*.*444 | 0*.*078 | 229 | 12 |
|  | 16-17 | 0*.*053 | 0*.*618 | 0.612 | 0*.*750 | 0*.*164 | 209 | 11 |
|  | 17-18 | 0*.*046 | 0*.*645 | 0.651 | 0*.*350 | 0*.*037 | 190 | 8 |
|  | 18-19 | 0*.*069 | 0*.*539 | 0.542 | 0*.*429 | 0*.*040 | 174 | 11 |
|  | 19-20 | 0*.*065 | 0*.*803 | 0.818 | 0*.*125 | 0*.*016 | 160 | 5 |
|  | 20-21 | 0*.*064 | 0*.*484 | 0.491 | 0*.*429 | 0*.*038 | 148 | 10 |
|  | 21-22 | 0*.*063 | 0*.*793 | 0.814 | 0*.*400 | 0*.*078 | 132 | 6 |
|  | 22-23 | 0*.*105 | 0*.*140 | 0.151 | 0 | 0 | 115 | 1 |
|  | 23-24 | 0*.*012 | 0*.*215 | 0.209 | 0*.*500 | 0*.*011 | 108 | 2 |
|  | 24-25 | 0*.*064 | 0*.*477 | 0.458 | 0*.*583 | 0*.*075 | 101 | 7 |
|  | 25-26 | 0*.*029 | 0*.*481 | 0.499 | 0 | 0 | 90 | 3 |
|  | 26-27 | 0*.*062 | 0*.*546 | 0.542 | 0*.*250 | 0*.*037 | 79 | 5 |
|  | 27-28 | 0*.*069 | 0*.*723 | 0.733 | 0*.*500 | 0*.*083 | 68 | 4 |
|  | 28-29 | 0*.*083 | 0*.*610 | 0.602 | 0*.*625 | 0*.*093 | 59 | 5 |
|  | 29-30 | 0*.*056 | 0*.*595 | 0.620 | 0*.*500 | 0*.*071 | 45 | 2 |
|  | **mean** | **0.056** | **0.556** | **0.560** | **0.383** | **0.043** |  |  |
| Three-year |  |  |  |  |  |  |  |  |
|  | 10-13 | 0*.*019 | 0*.*614 | 0.614 | 0*.*600 | 0*.*020 | 277 | 5 |
|  | 13-16 | 0*.*083 | 0*.*468 | 0.464 | 0*.*417 | 0*.*070 | 252 | 22 |
|  | 16-19 | 0*.*140 | 0*.*523 | 0.509 | 0*.*632 | 0*.*168 | 209 | 30 |
|  | 19-22 | 0*.*150 | 0*.*655 | 0.717 | 0*.*194 | 0*.*220 | 160 | 21 |
|  | 22-25 | 0*.*088 | 0*.*444 | 0.444 | 0*.*429 | 0*.*056 | 115 | 10 |
|  | 25-28 | 0*.*141 | 0*.*610 | 0.622 | 0*.*438 | 0*.*152 | 90 | 12 |
|  | 28-31 | 0*.*148 | 0*.*689 | 0.706 | 0*.*611 | 0*.*226 | 59 | 8 |
|  | **mean** | **0.110** | **0.572** | **0.582** | **0.474** | **0.130** |  |  |
| Five-year |  |  |  |  |  |  |  |  |
|  | 10-15 | 0*.*053 | 0*.*519 | 0.523 | 0*.*458 | 0*.*036 | 277 | 15 |
|  | 15-20 | 0*.*199 | 0*.*581 | 0.581 | 0*.*577 | 0*.*272 | 229 | 47 |
|  | 20-25 | 0*.*177 | 0*.*641 | 0.688 | 0*.*427 | 0*.*238 | 148 | 26 |
|  | 25-30 | 0*.*215 | 0*.*662 | 0.667 | 0*.*600 | 0*.*350 | 90 | 19 |
|  | **mean** | **0.161** | **0.601** | **0.615** | **0.515** | **0.224** |  |  |

*Definition of sub-threshold clinically significant symptoms*

Clinically significant activation (hypomanic symptoms) was defined as follows: 1) participant endorsed a minimum of three DSM-IV hypomanic symptoms, but did not meet full DSM-IV criteria for hypomania or bipolar disorder not otherwise specified episode based on duration or severity; (2) symptoms represented a clear change from normal functioning, endorsed by self and others that knew the person well; and (3) no evidence of major impairment.

Clinically significant subthreshold depressive symptoms: participant endorsed a minimum of three DSM-IV depressive symptoms one of which included depressed mood but did not meet full criteria for a major depressive episode or depression not otherwise specified based on duration or severity; (ii) symptoms represented a clear change from normal functioning, endorsed by self and others that knew the person well; and (iii) no evidence of major impairment.

Clinically significant subthreshold anxiety symptoms: participant endorsed a minimum of three DSM-IV anxiety symptoms one of which was feeling anxious but did not meet full criteria for an anxiety disorder or anxiety not otherwise specified based on duration or severity; (ii) symptoms represented a clear change from normal functioning, endorsed by self and others that knew the person well; and (iii) no evidence of major impairment.

Clinically significant subthreshold sleep symptoms: participant endorsed a minimum of three DSM-IV sleep symptoms but did not meet full criteria for a sleep disorder diagnosis based on duration or severity; (ii) symptoms represented a clear change from normal functioning, endorsed by self and possibly by others that knew the person well; and (iii) no evidence of major impairment.

Clinically significant subthreshold substance use symptoms: participant endorsed a minimum of three DSM-IV substance abuse symptoms, but did not meet full criteria for a substance use disorder diagnosis; (ii) symptoms were a concern to self and/or others who knew the person well; (iii) no evidence of major impairment.

Table S4 presents the number of subthreshold diagnoses for individuals in the full data set, the individuals who experienced the outcome (bipolar spectrum disorder) during the follow-up period and the individuals who did not experience the outcome diagnosis by the end of the follow-up period.

**Table S4.**  Number of subthreshold diagnoses for individuals in the full sample, individuals who experienced the major mood disorder outcome (diagnosis of BP, MDD and/or schizoaffective disorder), and individuals who did not experience the outcome.

| Number of sub-threshold diagnoses | Full sample (n=292) | Individuals with outcome (n=112) | Individuals without outcome (n=180) |
| --- | --- | --- | --- |
|  | n (%) | n (%) | n (%) |
| 0 | 173 (59.2) | 58 (51.8) | 115 (63.9) |
| 1 | 91 (31.2) | 41 (36.6) | 50 (27.8) |
| 2 | 25 (8.6) | 12 (10.7) | 13 (7.2) |
| 3 | 3 (1.0) | 1 (0.3) | 2 (1.1) |
| ≥ 4 | 0 (0.0) | 0 (0.0) | 0 (0.0) |

Reference List

1. Cox DR. Regression models and life‐tables. Journal of the Royal Statistical Society: Series B (Methodological). 1972;34(2):187–202.
2. Efron B. Logistic regression, survival analysis, and the Kaplan-Meier curve. Journal of the American statistical Association. 1988;83(402):414–25.
3. Allison PD. Survival analysis using SAS: a practical guide. Sas Institute; 2010.
4. Kaplan EL, Meier P. Nonparametric estimation from incomplete observations. Journal of the American statistical association. 1958;53(282):457–81.
5. Warner B, Misra M. Understanding neural networks as statistical tools. The American Statistician. 1996;50(4):284–93
6. Biganzoli E, Boracchi P, Mariani L, Marubini E. Feed forward neural networks for the analysis of censored survival data: a partial logistic regression approach. Statistics in medicine. 1998;17(10):1169–86.
7. Ruder S. An overview of gradient descent optimization algorithms. arXiv preprint arXiv:160904747.

2016

1. Tibshirani R. Regression shrinkage and selection via the lasso. Journal of the Royal Statistical Society: Series B (Methodological). 1996; 58(1):267-88.
2. Antolini L, Boracchi P, Biganzoli E. A time‐dependent discrimination index for survival data. Statistics in medicine. 2005;24(24):3927–44.
3. Zhou X-H, Obuchowski NA, McClish DK. Measures of Diagnostic Accuracy. In: Statistical Methods in Diagnostic Medicine [Internet]. John Wiley & Sons, Ltd; 2011. p. 13–55. Available from: <https://onlinelibrary.wiley.com/doi/abs/10.1002/9780470906514.ch2>
4. Graf E, Schmoor C, Sauerbrei W, Schumacher M. Assessment and comparison of prognostic classification schemes for survival data. Statistics in medicine. 1999;18(17‐18):2529–45.
5. Chu K. An introduction to sensitivity, specificity, predictive values and likelihood ratios. Emergency Medicine. 1999;11(3):175–81.
6. Youden WJ. Index for rating diagnostic tests. Cancer. 1950;3(1):32–5.
7. Kohavi R. A study of cross-validation and bootstrap for accuracy estimation and model selection. In: Ijcai. Montreal, Canada; 1995. p. 1137–45.
